# A French Hospital: An Inside View

**Published:** 1887-08-27

**Authors:** 


### A French Hospital: an Inside View.

By an Englishman and Former Patient.

"Live and Learn."

With the proud heart of Englishmen, we are apt to look askance at foreign institutions, and persuade ourselves in our ignorance that our system for this or that is as perfect as human ingenuity can make it. In the matter of hospitals I certainly think we can, in some respects at least, take a lesson out of the Frenchman's book, though we may not be the advocates of a principle by which charity is, to a certain extent, stifled through the State or the civic municipality stepping in to the support of the institution. But, in the instance which I am about to quote, the nobility of charity is far from being an unknown factor.

The Hopital St. Andre.

I must ask my readers mentally to accompany me to the "sunny south" of France—to the wine-producing province of the Gironde. There, in its capital, Bordeaux, the far-famed *Burdigala* of the *Bituriges Vivisci*, we will for a few minutes stay. The Hôpital St. André, to which I invite attention, is a gigantic building, larger than any but the largest London hospital. It has a grand total of eight hundred beds, nearly the whole of which are always full. Bordeaux is a great and populous city, and its principal branches of industry—the production or preparation of sugar, brandy, vinegar, printed calicoes, woollen goods, paper, etc.—afford sustenance to a very large percentage of workers, for whom the Hôpital St. André is the great refuge in the hour of sickness. The hospital itself is a somewhat handsome building, two storeys in height, and quadrangular in design. The space enclosed by the wards consists of a spacious square laid out as a garden. The warm climate of Bordeaux affords the patients who are sufficiently well, frequent opportunities to promenade in the stone galleries, which command a full view of the gardens, in the centre of which is a large fountain. The grounds are kept in exquisite order, but, except on very rare occasions, the patients are not allowed to go into them.

Its Benefactors.

The Hôpital St. André appears to have had some very generous benefactors, especially an Englishman, one Nathaniel Johnston, who founded the well-known wine firm of that name. Walking round the galleries to which I have alluded, the visitor will see sundry tablets against the walls, and a perusal of them will give the observer a very fair idea of the munificence which this mighty asylum for the sick poor has met with. The internal management of this hospital compares very favourably with that of the great hospitals of London. The rooms are admirably ventilated, and scrupulously clean. The nursing is, as is the custom in France, done by Sisters of Charity. There are crucifixes and other symbols of the Catholic faith in every ward, and of course the vast mass of the patients are of the Romish creed; but, should a Protestant or a person of any other religious belief come within the walls of the Hôpital St. André, there is no attempt made to win him from his faith. The sisters have, most of them, devoted their lives to the noble profession of succouring the sick, and some of those whom one will see attending the wards are women of quite advanced years. It is very pleasing to observe the zeal, the earnestness, the love, the absolute devotion of these good women, whose lives are dedicated to God, whose hearts are dedicated to the suffering poor. Kindness to the sick, the aged, and the infirm has always been a distinguishing attribute of the Catholic faith, and nowhere is it seen in greater fulness than within the walls of a great French hospital.

Paying Patients and State Aid.

At the Hôpital St. André patients have to pay according to their means. This does not mean that everybody has to pay, or that everybody who pays has to contribute the whole of his cost. In America, where there is a capital system of pay hospitals, they have what are known as the "remunerative" and the "unremunerative" pay patient. It is so at Bordeaux. "Small" and "large" payments are taken. The "small" pay patients contribute 3*s*. 4*d*. a day, and the "large" pay patients from 5*s*. 10*d*. to 6*s*. 8*d*. All who are really ill are admitted upon some terms or another. With regard to State aid, the rule at the Bordeaux hospital is to receive elevenpence per diem from the municipal funds for each patient resident in the town itself; whilst for those using the hospital, but living outside the town, the townships contribute one shilling and threepence a day in each individual case. In England, at present, we have no hospital rate, and charity has its full play; but it seems to me that much of the chronic bankruptcy of our hospitals might be avoided if we developed the pay-system a little further. Where it has been tried it has, thus far, met with unqualified success, and I feel confident that the day is not far distant when it will be extended much further. "Welcome evermore to gods and men," writes Emerson, "is the self-helping man." The charity which reduces us to the level of the pauper is a social evil which we ought not to tolerate. Where there is absolute need of gratuitous relief, I say give it by all means; but it is a fact beyond dispute that nine-tenths of the applicants who fill our out-patient departments at the present time are people who are able to contribute, more or less, to the charity they receive.

